# Mapping open chromatin by ATAC-seq in bread wheat

**DOI:** 10.3389/fpls.2022.1074873

**Published:** 2022-11-16

**Authors:** Xin Wang, Chuanye Chen, Chao He, Dijun Chen, Wenhao Yan

**Affiliations:** ^1^ National Key Laboratory of Crop Genetic Improvement, Hubei Hongshan Laboratory, Huazhong Agricultural University, Wuhan, China; ^2^ State Key Laboratory of Pharmaceutical Biotechnology, School of Life Sciences, Nanjing University, Nanjing, China

**Keywords:** ATAC-seq, chromatin accessibility, reproducible peak, data evaluation, regulatory elements

## Abstract

Gene transcription is largely regulated by *cis*-regulatory elements. Assay for Transposase-Accessible Chromatin using sequencing (ATAC-seq) is an emerging technology that can accurately map *cis*-regulatory elements in animals and plants. However, the presence of cell walls and chloroplasts in plants hinders the extraction of high-quality nuclei, thereby affects the quality of ATAC-seq data. Meanwhile, it is tricky to perform ATAC-seq with different tissue types, especially for those with limited size and amount. Moreover, with rapid growth of ATAC-seq datasets from plants, powerful and easy-to-use data analysis pipelines for ATAC-seq, especially for wheat is lacking. Here, we provided an all-in-one solution for mapping open chromatin in wheat including both experimental and data analysis procedure. We efficiently obtained nuclei with less cell debris from various wheat tissues. High-quality ATAC-seq data from young spike and ovary, which are hard to harvest were generated. We determined that the saturation sequencing depth of wheat ATAC-seq is about 16 Gb. Particularly, we developed a powerful and easy-to-use online pipeline to analyze the wheat ATAC-seq data and this pipeline can be easily extended to other plant species. The method developed here will facilitate plant regulatory genome study not only for wheat but also for other plant species.

## Introduction

*Cis*-regulatory elements, such as promoters, enhancers and insulators recruit transcription factors to form protein-DNA complexes, which regulate the transcription of downstream genes (Yan et al., 2019). DNA wraps around histone octamer to form a nucleosome (Kornberg, 1974; Luger et al., 1997; Richmond and Davey, 2003). There are naked DNA segments between nucleosomes, which are called open chromatin regions (OCRs). OCRs contain *cis*-regulatory elements and these regions are highly sensitive to endonucleases such as DNase I or transposases (Gross and Garrard, 1988).

Various techniques have been invented to estimate chromatin accessibility, namely Formaldehyde-Assisted Isolation of Regulatory Elements sequencing (FAIRE-seq), Deoxyribonuclease I (DNase I)-hypersensitive site sequencing (DNase-seq) and Assay for Transposase-Accessible Chromatin using sequencing (ATAC-seq) (Giresi et al., 2007; Song and Crawford, 2010; Buenrostro et al., 2013). The chromatin was sonicated randomly without preference in FAIRE-seq. It is convenient but the signal-to-noise ratio is low and the reproducibility is poor (Giresi and Lieb, 2009). DNase-seq maps DNase I-hypersensitive sites (DHSs) using DNase I that tends to cleave DNA fragments that are not occupied by nucleosomes or proteins. It is of high signal-to-noise ratio, but requires a large amount of tissues and the protocol is technologically tricky to handle (Tsompana and Buck, 2014). Tn5 is a bacterial transposase with high DNA cleavage activity. After incubation with DNA adapters, it can cut naked DNA and integrate the adapters into the target sites thus allows easy library preparation by PCR. Based on the feature of Tn5, ATAC-seq was developed to detect OCRs (Goryshin and Reznikoff, 1998; Gangadharan et al., 2010; Adey et al., 2010; Buenrostro et al., 2013). Benefit from its low nuclei input, handy library preparation and high signal-to-noise ratio, ATAC-seq is now widely applied in eukaryotic cells, including plants (Maher et al., 2018; Lu et al., 2019).

Since ATAC-seq is a nuclei based technology, high-purity and intact nuclei are required. In plant, it is challenging to obtain complete and pure nuclei. Existing of cell walls and various types of organelles represented by chloroplasts and mitochondria, and different cellular structures, such as root hairs and starch granules in endosperm largely affect the purity of plant nuclei (Weston et al., 2012; Okada et al., 2018). Since the organelle DNA is not protected by nucleosomes, it is arbitrarily cleaved by Tn5 transposase, resulting in the high rate of organelle contamination. Flow cytometry had been introduced to remove the impact of organelles and other undesired structures in *Arabidopsis* and rice (Lu et al., 2017; Zhu et al., 2020).

Analysis of ATAC-seq data is hard for labs without bio-informatics facility. To get high-quality, consistent, and reproducible OCRs, the Encyclopedia of DNA Elements (ENCODE) Data Coordinating Center Uniform has proposed a standard ATAC-seq data processing pipeline, which considers alignment rate, library complexity, peak number, insert fragment length, the fraction of reads in called peak regions (FRiP) score and transcription start site (TSS) enrichment to evaluate the data quality (Sloan et al., 2016). Nevertheless, the ENCODE ATAC-seq pipeline was designed for human and animals. Since plant cells contain cell walls and chloroplasts, so the parameters and criteria to evaluate data quality should be adjusted accordingly, especially for wheat with a complex polyploidy genome.

Bread wheat contains three sub-genomes and has a gigantic genome with more than 93% non-coding regions and extremely low gene density (Ramirez-Gonzalez et al., 2018; Zhu et al., 2021). It is highly demanded to obtain open chromatin information for the exploration of functional *cis*-regulatory elements from the complicated genome in wheat. Currently, there are a few studies to investigate chromatin accessibility in wheat. Moussa Benhamed and colleagues mapped open chromatin in wheat seedlings by ATAC-seq and found that the intergenic condensed spacer (ICONS) border is highly accessible in wheat, while the internal ICONS accessibility is reduced (Concia et al., 2020). The chromatin status of the 9-day old seedlings had also been reported (Li et al., 2019). Among all these studies, they either applied other methods than ATAC-seq to map open chromatin or only the leaf tissue was used. Leaf is one of the most common tissue types to sample. However, there are quite some tissue-types such as young inflorescence and early embryo are not easy to harvest. For these tissue types, ATAC-seq is an optimal method to map open chromatin since it needs only small number of nuclei. So, it is of particular interest to establish an efficient ATAC-seq protocol for various tissue types in wheat.

Here, we optimized nuclei extraction protocol with specific attention to buffer condition for different tissue types in wheat. Flow cytometry was used to further purify the nuclei. Then, the purified nuclei were submitted to Tn5 tagmentation and library preparation. We used this protocol to map open chromatin of young spike at double-ridge stage and ovary at 2 days after pollination, two types of tissues that are hard to harvest. Furthermore, we also developed pipeline to analyze ATAC-seq data in wheat.

## Materials and methods

### Plant growth and sample harvesting

The bread wheat variety Aikang 58 (AK58) was used in this study. Seeds were sterilized in 75% alcohol and then washed three times with water. Seeds were germinated on filter paper at 25°C under 16/8 light/dark conditions. Two days later, the radicles were harvested and the remaining part was transferred to the soil. The plants were grown at 22°C in greenhouse. We harvested young spike at double-ridge stage and ovary at 2 days after pollination. Half of the above materials were stored at -80°C for RNA-seq and the other half for nuclei extraction.

### Extraction of crude nuclei

Except the double-ridge stage young spike which had been stored at -80°C for more than 1 month, fresh material was collected and was immediately chopped in any of D-S-0.4 buffer (0.4 M D-Sorbitol, 20 mM MES, 20 mM KCl, 10 mM MgCl_2_, 3 mM DTT, 0.4% Triton X-100 1% BSA, 1 tablet/50 ml protease inhibitor), D-Sorbitol buffer (0.6 M D-Sorbitol, 20 mM MES, 20 mM KCl, 10 mM MgCl_2_, 1 mM β-mercaptoethanol, 1×PMSF buffer, 0.2% TritonX-100 0.1 mg/ml BSA, 1 tablet/50 ml protease inhibitor), Chopping Buffer (15 mM Tris-HCl, 20 mM NaCl, 80 mM KCl, 0.5 mM spermine, 5 mM 2-ME, 0.4% TritonX-100) or PP Buffer (1×PBS containing 1 tablet/50 ml protease inhibitor, 0.4% TritonX-100). After chopping, the liquid was filtered with 30 μm strainer.

### Nuclei sorting and ATAC-seq assay

Crude nuclei were stained with DAPI and loaded into a flow cytometer (BD FacsAria II SORP). 50,000 nuclei were sorted into 400 μl buffer. The nuclei were collected by centrifugation at 1,000 g for 10 min at 4°C. After removing the supernatant but 20 μl liquid remained, premixed 1×TD buffer (10 mM Tris-HCl, 5 mM MgCl_2_, 10% v/v DMF) and 1.5 μl TTEMix V50 (Vazyme, #TD501) were added to the nuclei solution and incubated at 37°C for 30 min. The reaction was terminated and DNA was purified using the QIAGEN MinElute PCR Purification Kit (QIAGEN, #28006). The eluted DNA was used to prepare library with either TruePrep DNA Library Prep Kit V2 (Vazyme, #TD501) or NEBNext High-Fidelity2X PCR Master Mix (NEB, M0541). The primer sequences are listed in **Table S1**. The precise number of PCR cycles was determined by qPCR (Bajic et al., 2018). The libraries were purified with AMPure beads. The concentration of ATAC-seq libraries were measured using Qubit. The fragment sizes of the library were calculated by Agilent 2100 Bioanalyzer. The libraries were sequenced by the NovaSeq 6000 system with the 150-bp paired-end mode.

### RNA-seq assay

Tissue was snap-frozen in liquid nitrogen and stored at -80°C. Total RNA from young spike at double-ridge stage and ovary at 2 days after pollination was extracted using Trizol (Ambion, #15596018). The concentration and purity of RNA were detected by Nanodrop (Thermo Fisher Scientific) and electrophoresis. RNA-seq libraries from the two tissues were prepared using the VAHTS^®^ Universal V8 RNA-seq Library Prep Kit for MGI (Vazyme, #NRM605). RNA-seq libraries were sequenced by DNBSEQ-T7 (BGI, Shenzhen) with the 150-bp paired-end mode. RNA-seq was performed in triplicates.

### Analysis of ATAC-seq data

The quality of raw sequencing data was estimated using Trimmomatic v0.32 (https://github.com/usadellab/Trimmomatic) with parameters of “LEADING: 20 TRAILING: 20 SLIDINGWINDOW: 4: 15 MINLEN: 36”. The clean data was mapped to IWGSCv1.0 using bowtie2 v2.4.4 with parameters “–sensitive -k 3” (Langmead et al., 2009). After removing PCR duplicates using Picard v2.23.9 and low-quality reads (Filter unmatched & MAPQ < 5 reads) using SAMtools v1.9 (Li et al., 2009), the high quality clean reads were analyzed using MACS2 v2.2.7.1 (Zhang et al., 2008) with the following parameters: –nomodel –shift -75 –extsize 150 –to-large -B –SPMR. The shift length is our empirical value after hundreds of predictions using phantompeakqualtools (https://github.com/kundajelab/phantompeakqualtools). In order to remove false positive peaks, we used IDR to get the consistent peaks, which was recommended by ENCODE. The FRiP and the SPOT coefficients were used to evaluate the signal-to-noise ratio of the libraries. The final bam files produced from reads alignment were converted to bigwig format for visualization on genome browser using bamCoverage of deepTools v3.5.0 (Ramirez et al., 2016). The correlation between samples were calculated and profiled by the multiBigwigSummary and plotCorrelation command of deepTools toolkit, respectively. Generally, the Pearson Correlation Coefficient greater than 0.8 is regarded as better correlation. Further, the deepTools computeMatrix command was used to analyze the signal density around the TSS and TES with the following parameters: computeMatrix scale-regions -p 10 -b 3000 -a 3000 -m 5000 –skipZeros. The distance from the peak summit to gene TSS was calculated by BEDTools v2.27, and then the locations of peaks were divided into genebody, promoter, intergenic upstream and downstream region using our developed R script. Genes with at least one specific peak within 4 kb upstream and 1.5 kb downstream of TSS were labeled as chromatin accessibility-related genes. ClusterProfiler was used to perform GO analysis with the obtained OCRs related genes (*q-*value cutoff = 0.05) (Wu et al., 2021). We developed a pipeline, called wheatATAC to analyze ATAC-seq data in wheat (https://github.com/hcph/wheatATAC.git).

### Calculation of saturated sequencing depth

We randomly extracted 10%, 20%, 30%, 40%, 50%, 60%, 70%, 80%, 90%, 100% and virtual 110%, 120%, 130%, 140%, 150%, 160%, 170%, 180%, 190% and 200% of the high quality unique mapped clean reads *via* SAMtools toolkit combined with Linux split command and performed peak calling *via* MACS2 (Zhang et al., 2008; Li et al., 2009). The saturated sequencing depth was determined *via* the saturationPlot function of the R-package, ATACseqQC (Ou et al., 2018).

## Results

### Optimization of nuclei extraction from different tissue types in wheat

**Figure 1:**
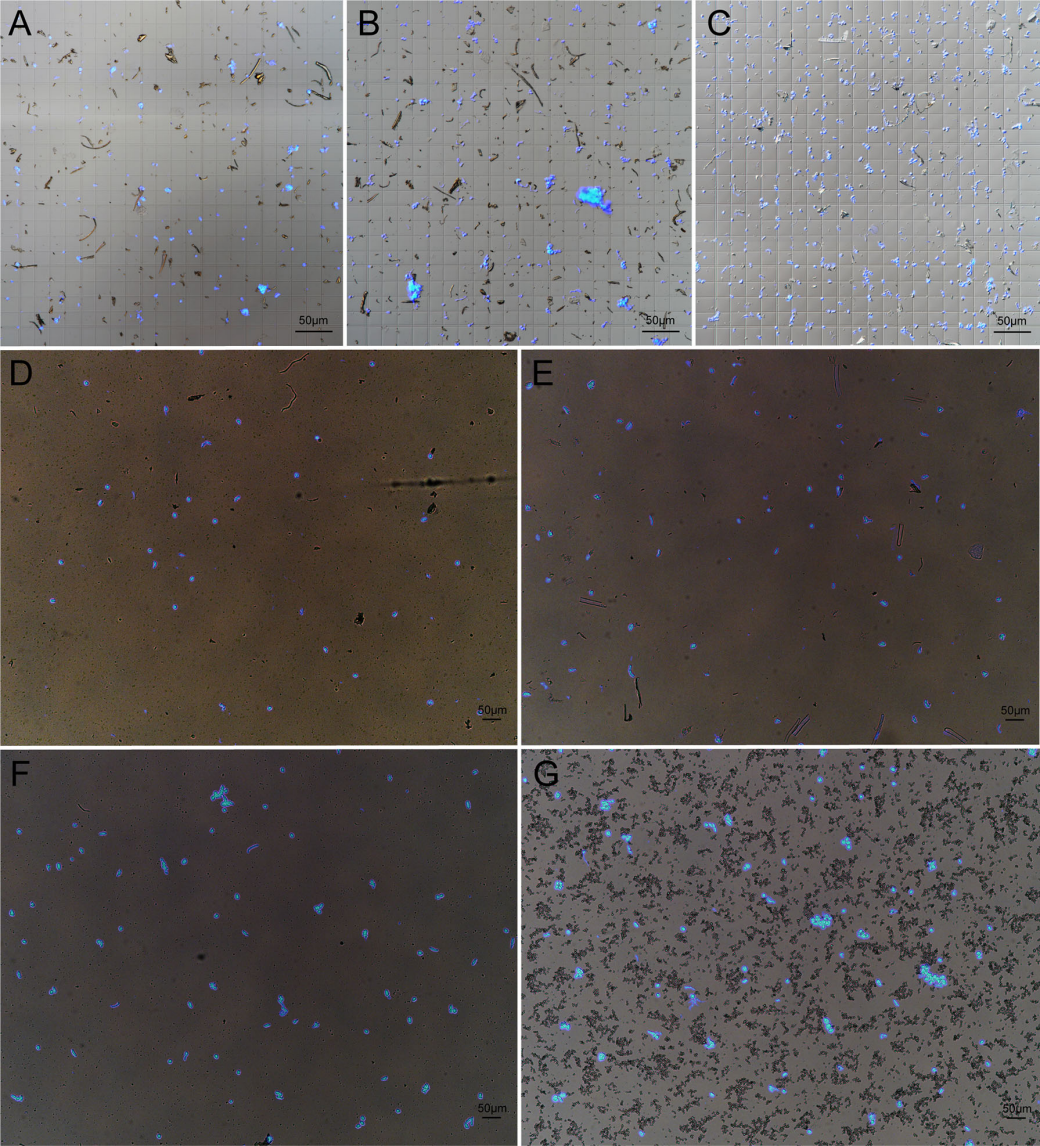
Extraction of high quality nuclei in wheat. Nuclei were extracted from 10-day old wheat seedlings by using PP Buffer **(A)**, Chopping Buffer **(B)** and D-Sorbitol Buffer **(C)**. A slightly modified D-S-0.4 Buffer was used to extracted nuclei from wheat leaf **(D)**, root **(E)**, young spike **(F)** and ovary at 4 d after pollination **(G)**. The nuclei solution was treated with 0.25 mg/L DAPI and 5 μl solution was taken for checking the quality of nuclei under fluorescent microscope.

Since grinding in liquid nitrogen is not suitable for nuclei extraction of small tissues, we chopped the tissue with blade for homogenization. In order to fix the suitable buffer condition to isolate nuclei in wheat, we tested three frequently used plant nuclei extraction buffers, PP buffer and D-Sorbitol buffer that worked nicely for maize (Tu et al., 2020; Sun et al., 2020), chopping buffer which had been widely used to extract nuclei from various plants (Lu et al., 2017; Maher et al., 2018; Lu et al., 2019; Zhu et al., 2020). We found that PP buffer was of low efficiency and generated cell debris (**Figure 1A**). Chopping Buffer produced more nuclei but the nuclei tended to aggregate with lots of cell debris (**Figure 1B**). The highest quality wheat nuclei were obtained using D-Sorbitol Buffer, not only regarding the number of nuclei but also the cleanest background as compared with the other two buffers (**Figure 1C**). Considering that high concentration of D-Sorbitol may affect later Tn5 tagmentation, we decreased the concentration of D-Sorbitol to 0.4 M (D-S-0.4). We then verified the applicability of D-S-0.4 buffer in different tissues, including aboveground part of 10-day old seedling, radical at 2 days after germination, double-ridge stage of spike (about 2 mm), and ovary at 4 days after pollination. As shown, the nuclei from different tissues are intact, which indicated that the D-S-0.4 buffer was suitable for nuclei extracting from different wheat tissues. However, different cellular structures and tissue specific components can be found in the suspension, such as root hairs from root tissue and starch grains from the ovary, but in general, cleaner nuclei could be obtained from tissues with meristematic activity, such as young inflorescence (**Figures 1D-G**).

### Establishing FANS-ATAC-seq method with low-input materials

**Figure 2:**
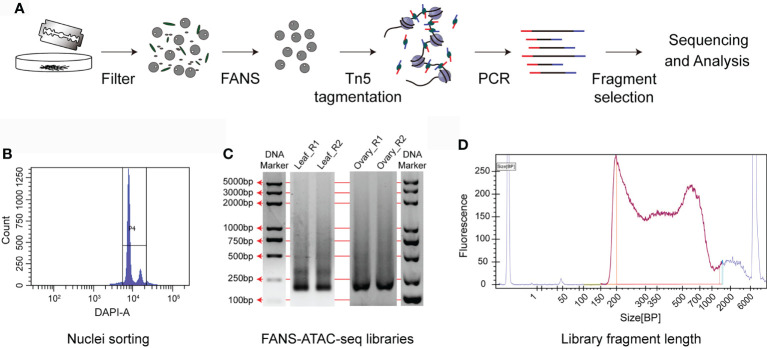
FANS-ATAC-seq protocol. **(A)** A schematic diagram of the FANS-ATAC-seq method, including nuclei preparation, sorting of nuclei and library construction. **(B)** P4 plot for the aboveground samples of 10-day old seedlings on flow cytometer. The nuclei sorting protocol was established based on the event number and DAPI fluorescence intensity. **(C)** Merged agarose gel picture to show the representative ATAC-seq libraries obtained after FANS. Discrete bands representing the nucleosome fractions indicate high quality of FANS-ATAC-seq libraries. Leaf_R1 and Leaf_R2 represent the two repeats of aboveground samples of 10-day old seedling, Ovary_R1 and Ovary_R2 represent the two repeats of ovary at 2 days after pollination. **(D)** Distribution of fragment length of the libraries for young spike at double-ridge stage.

In plant cells, besides the DNA fragments with open chromatin in the nuclei, there are exposed organelle DNA in mitochondria and chloroplasts, which can be cleaved by Tn5. Fluorescence activated nuclei sorting (FANS) sorts stained nuclei to get rid of other organelles. Combination of FANS with ATAC-seq (FANS-ATAC-seq) reduced the alignment rate of chloroplasts and mitochondria to 30% in *Arabidopsis* (Lu et al., 2017). To develop ATAC-seq protocol in wheat, we used simple chopping method followed by flow cytometry sorting to purify nuclei for Tn5 tagmentation (**Figure 2A**). In this method, the nuclei were stained with 4, 6-Diamidino-2phenylindole (DAPI) and then submitted to FANS. The nuclei can be divided into peaks of 2C and 4C. 2C represents the nuclei from somatic cells and 4C is for the dividing nuclei. We collected all the nuclei of 2C and 4C (**Figure 2B**). A total of 50,000 sorted nuclei were obtained for Tn5 tagmentation at 37°C for 30 min. The sequencing tag was inserted into the open chromatin through Tn5 and then the DNA was purified for library preparation. After tagmentation and PCR amplification, clear nucleosome pattern with different fragment sizes can be seen in the ATAC-seq libraries of aboveground of 10-day old seedling, ovary at two days after pollination and young spike at double-ridge stage. (**Figures 2C–D**), indicating proper digestion of Tn5. In order to evaluate the feasibility of FANS-ATAC-seq in wheat, especially with low amount of tissue input, we selected young spike at double-ridge stage and ovary at two days after pollination to generate ATAC-seq data (**Figure S1**). Only with ten spikes and ten 2-day old ovaries, we successfully got more than 300,000 nuclei and 50,000 were used for subsequent experiment.

### Saturation of sequencing depth for ATAC-seq library in wheat

**Figure 3:**
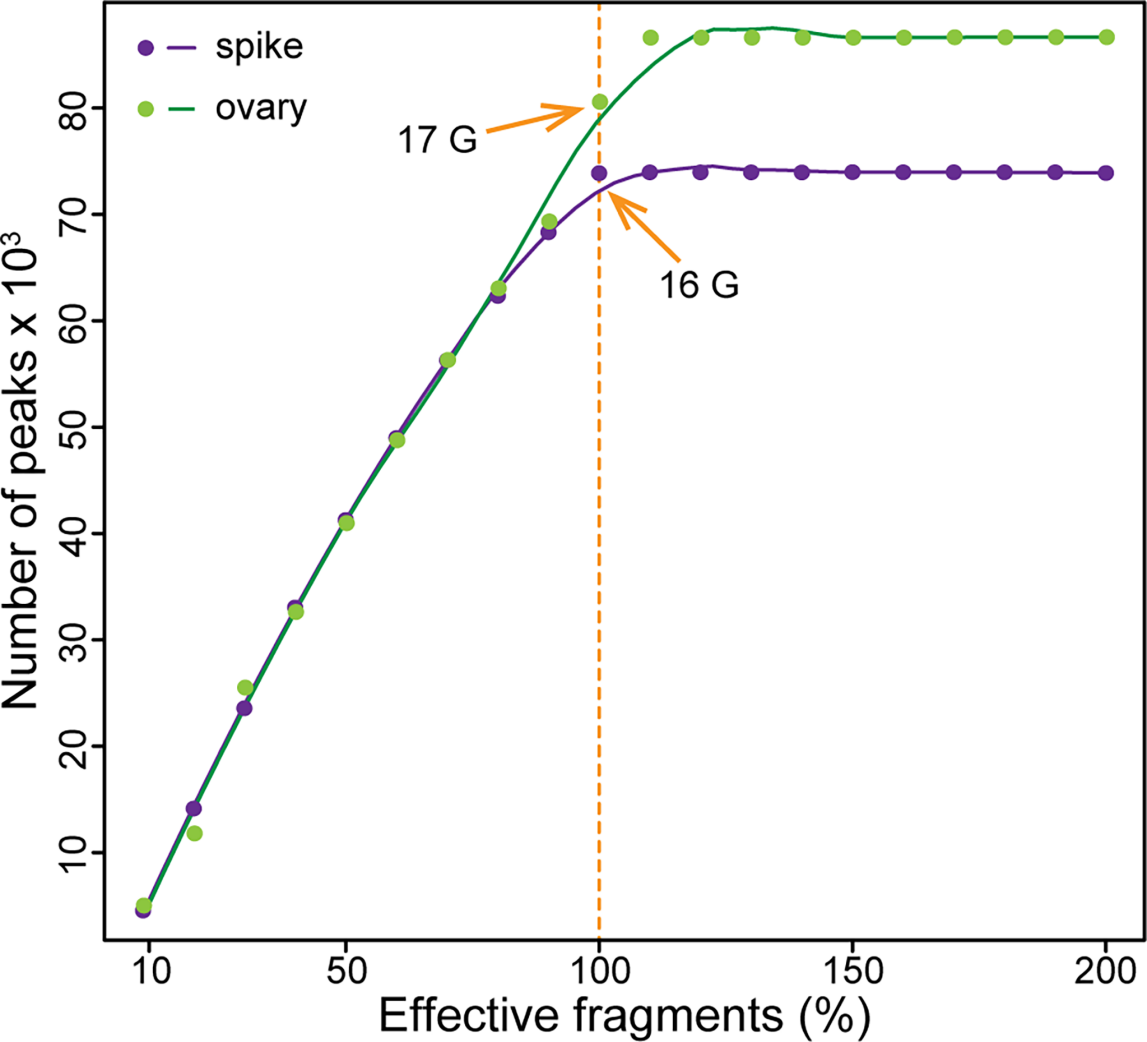
Sequencing saturation for ATAC-seq in wheat. The orange arrows indicate the saturated sequencing depth for ATAC-seq in wheat. The purple dots represent the peak numbers of double-ridge stage young spike at different sequencing depths, and the green dots represent that of ovary at 2 days after pollination.

How many reads are needed is always a question for next-generation sequencing based methods. The sequencing depth is quite important for subsequent qualitative and quantitative analysis. Insufficient sequencing data may cause false positive results and lost positive signals due to the interference from background noise. Exceeded sequencing not only wastes money, but also reduces the signal-to-noise ratio. To explore the sequencing saturation of ATAC-seq in wheat, we sequenced one library from each of the young spike and the ovary for 16-17 Gb high quality clean data, which is similar to the size of wheat genome. Then, we randomly extracted from 10% to 200% of the reads from the clean data to call peaks, respectively. The results show that the saturation of sequencing depth is sample dependent, but in general, 16-17 Gb data is optimal for ATAC-seq in wheat (**Figure 3**). Thus, we aimed for 16 Gb data for each of the four libraries.

### Development of pipeline for ATAC-seq data analysis in wheat

**Figure 4:**
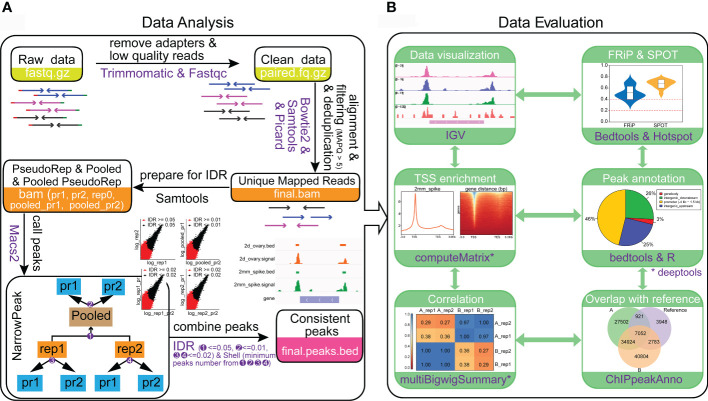
Protocol for ATAC-seq data analysis and data quality evaluation. **(A)** Steps for ATAC-seq data analysis of wheat. **(B)** Processes and metrics to evaluate the data quality of ATAC-seq. The results were generated from the young spike and ovary samples with two biological replications. A_rep1 and A_rep2 represent the two replications from young spike. B_rep1 and B_rep2 are the two replications from ovary. The reference in the venn diagram represents the open chromatin regions obtained from GSM3564343.

ATAC-seq data analysis is challenging in wheat due to the presence of more than 80% repetitive sequences in the genome. We established a workflow and a pipeline to analyze ATAC-seq datasets in wheat and it can be easily extended to other plant species. To solve the problem of high ratio of repetitive sequences in wheat, we set the number of allowed mismatches to 0 when aligning the clean reads to the reference genome of IWGSCv1.0. The imperfectly aligned reads and reads from PCR duplication were removed. By using Bowtie2 for alignment and the “SAMtools index -c” command to build a csi index for bam files, the reads can be aligned directly to the reference genome without splitting the chromosomes, thus avoiding the hassle of position conversion. To reduce the false positive rate and obtain the high-quality, consistent, and reproducible peaks, the Irreproducible Discovery Rate (IDR) values between original replicates, the pseudo-replicates which respectively and randomly split from each of the original replicate, and the pseudo-replicates that randomly split after pooling the replicates were calculated by IDR v2.0.4.2 (https://github.com/nboley/idr), respectively (**Figure 4A**). The IDR value of 0.05, 0.02 and 0.01 are the recommended cutoffs for the original replicates, the pseudo-replicates split from original replicates and pooled pseudo-replicates, respectively for wheat ATAC-seq analysis. The whole procedure can be divided into five steps, including i) removing the adapters to obtain clean reads, ii) generation of unique mapped reads, iii) preparing files for IDR analysis, iv) calling peaks and v) combination of consistent peaks (**Figure 4A**).

A successful ATAC-seq should capture OCRs with high signal-to-noise ratio. In general, the most direct way for data estimation is to visualize peaks in genome browser like Integrative Genomics Viewer (IGV). We integrated the quality evaluation process in the pipeline by including the production of visualization files, calculation for library complexity, insert fragment distribution, sample cross correlation, TSS enrichment score, FRiP and the Signal Portion Of Tags (SPOT) values (**Figure 4B**). In addition, the functional feature of the peak region and the overlapping ratio of the peaks with other tissues are also indicators of data quality (**Figure 4B**). Both the data processing and date quality evaluation pipelines are available in Github (https://github.com/hcph/wheatATAC.git). The users can take raw sequence data as input to obtain OCRs and all the results for quality evaluation (**Figure 4B**). Since the parameters are easy to be adjusted, the pipeline is generally applicable to other plant species. Importantly, as the finally obtained signals would have been filtered by multiple comparisons, the open chromatin signals are reproducible and comparable across different libraries. In addition, the pipeline is based on Linux system and is highly flexible.

### Mapping open chromatin in young spike and ovary

**Figure 5:**
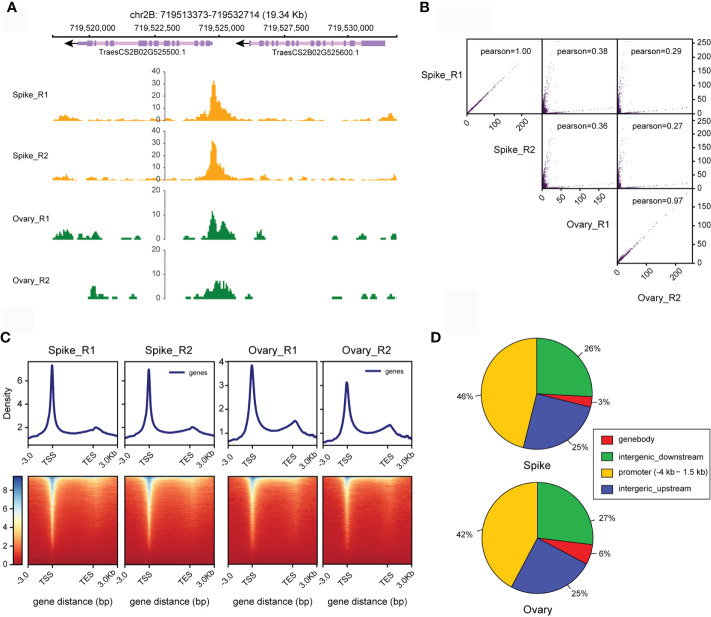
Estimation of the quality of ATAC-seq data from young spike and ovary. **(A)** Visualizing ATAC-seq data for young spike and ovary *via* IGV. **(B)** Scatter plot of the correlations between replicates. The Pearson correlation coefficient was calculated. **(C)** Distribution frequency of open chromatin regions in young spike and ovary at TSS and TES. **(D)** Proportions of distribution of open chromatin regions in the genome in young spike and ovary.

Using the data processing strategy we proposed, we found that at least 90% of the four datasets we obtained here from young spike and ovary can be aligned to the wheat genome with the highest genome alignment rate of 95% (**Table S2**). We then used these data to predict OCRs in young spike and ovary of wheat. After preformed IDR analysis, we finally obtained 70,766 and 86,041 OCRs in young spike and ovary, respectively. These OCRs are consistent among replications. The number of OCRs in ovary is higher than that in young spike. The signal-to-noise ratios of the ATAC-seq data from both tissues were high (**Figure 5A**). The correlation co-efficiency between the replication of tissues is higher than 0.97, especially in young spike, with a co-efficiency of almost 1.00 (R score), indicating the reproducibility of our ATAC-seq protocol (**Figure 5B**). Not surprisingly, chromatin accessibility between young spike and ovary is lowly correlated.

We defined the 4 kb upstream to 1.5 kb downstream of TSS as promoter region and the regions beyond 4 kb upstream of TSS or downstream of transcription end site (TES) were treated as intergenic regions where distal regulatory elements like enhancers may exist. The distribution of the predicted OCRs in TSS and TES showed the same trend in young spike and ovary (**Figure 5C**). This is consistent with the result in *Arabidopsis thaliana* (Lu et al., 2017). However, different from the situation in *Arabidopsis*, more than 50% of the open chromatin can be found in intergenic region in wheat, which might indicate the presence of distal regulatory elements in these regions or it might be due to the super large genome size with large number of repetitive sequences and more complex spatial genome organization in wheat (**Figure 5D**).

### Gene activity is highly co-related with chromatin accessibility

**Figure 6:**
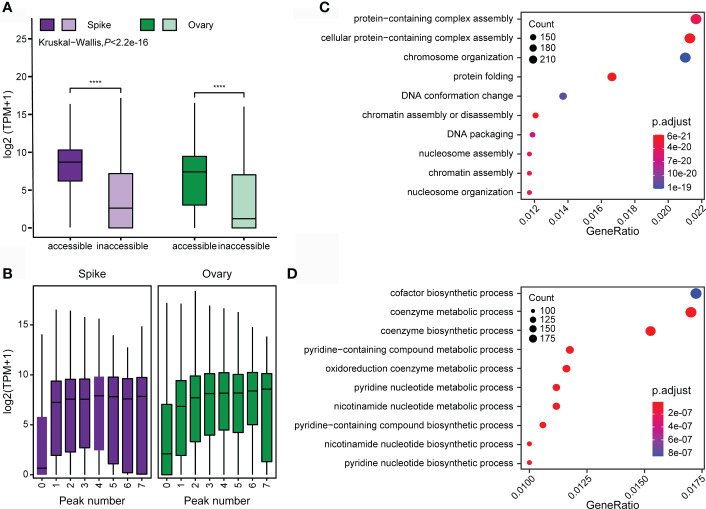
Correlation between chromatin accessibility and gene transcription in young spike and ovary. **(A)** Differences in gene transcription level between the genes with and without open chromatin. **** indicates P < 0.0001. **(B)** Correlation between gene transcription level and the number of ATAC-seq peaks. GO enrichment analysis of chromatin accessibility-related genes in young spike **(C)** and ovary **(D)**.

A high correlation between chromatin accessibility and gene activity is expected since OCRs are more preferable to be bound by regulatory proteins to alter target gene transcription. In total, 29,909 genes in young spike and 33,452 genes in ovary were identified with ATAC-seq peaks in the promoter regions. We profiled the gene transcription of young spike and ovary. The transcription level of genes with ATAC-seq peaks is significantly higher than those without any ATAC-seq peaks (**Figure 6A**). Strikingly, the expression level is directly proportional to the number of peaks (**Figure 6B**).

We performed GO analysis on the relevant genes obtained from the tissue-specific ATAC-seq peaks of young spike and ovary, respectively. Genes with OCRs in young spike are mainly clustered in the pathways of protein-containing complex assembly, chromosome organization, protein folding and DNA conformation change (**Figure 6C**), which suggests that open chromatin mediates rapid cell division and differentiation in young spike by affecting protein complex assembly and DNA conformation. Most of the open chromatin related genes in ovary are in the coenzymes synthesis and nucleotide metabolism pathways (**Figure 6D**), indicating active metabolic reactions in the ovary. All these results above again approved the high quality of our ATAC-seq data from the aspect of biological sense.

## Discussion

The ATAC-seq method, first reported in mammals, is an ideal replacement of DNase-seq and FAIRE-seq to explore chromatin accessibility because of its short library preparation time and high signal-to-noise ratio, especially the great potential in single cell analysis (Buenrostro et al., 2015; Zhang et al., 2021). Here, we report an efficient and tissue type independent ATAC-seq protocol in wheat with low-input.

Obtaining complete and pure nuclei is the key to the success of ATAC-seq experiments. Using different buffers to extract maize nuclei, the results show that the efficiency of extracting nuclei is different, and the nuclei extracted with different buffers have obvious cytological differences (Vera et al., 2014). Similar result occurred in wheat nuclei extraction here. Compared with a similar FANS based ATAC-seq protocol, our protocol here optimized tissue homogenization and nuclei extraction steps, which led to higher production and purity of nuclei. We saw only around 5% organelle contaminations, compared with 30% reported previously (Lu et al., 2017). By using the protocol development here, the genome alignment rate and organelle contamination rate are perfectly controlled. Since both D-Sorbitol buffer and FANS had been used for other species (Lu et al., 2017; Tu et al., 2020), we expected that the ATAC-seq protocol developed here would have broad applicability.

Particularly, we proposed a pipeline for data analysis in wheat. A standardized pipeline to analyze ATAC-seq data had been proposed by the ENCODE Data Coordination Center Uniform mainly for human and animals (Sloan et al., 2016). In the present study, we have well evaluated and chosen the suitable analysis tools as well as the corresponding parameters for plant specific ATAC-seq data analysis. The pipeline is efficiently working for large genome like wheat and all the parameters can be easily adjusted for other species if needed. We set the maximum allowed mismatch number to 0 to increase the mapping accuracy. By using csi index, there is no need to split each chromosome into two parts as been done by the ATACseqMappingPipeline (https://github.com/lufuhao/ATACseqMappingPipeline). Two metrics namely FRiP and SPOT values are representative for signal-to-noise ratio of the sequencing data. The FRiP value is calculated based on the number of finally obtained peaks. The SPOT uses the binomial distribution with a local background model to automatically correct for broad-scale regional differences in tag levels; therefore, it is more suggestive than FRiP. However, we recommend using both the FRiP and the SPOT value to evaluate the data quality since FRiP could indicate the efficiency of Tn5 tagmentation. Active transcription is highly correlated with open chromatin, thus chromatin is more likely to be open at the TSS (Miyamoto et al., 2018). Given that the promoter of actively transcribed gene should be accessible by upstream regulators and also the fact that more than 93% of the wheat genome is non-coding regions (Ramirez-Gonzalez et al., 2018), it is expected that most of the OCRs are located in the gene promoter and intergenic regions. Notably, higher number of peaks does not mean higher quality of the data. All of the parameters mentioned above should be collectively considered to draw conclusions. Moreover, the predicted OCRs generally correlate well with gene expression levels.

We obtained high-quality ATAC-seq data of young spike at the double-ridge stage and ovary at 2 days after pollination, providing data supports for exploring changes in chromatin accessibility, nucleosome occupancy, and tissue-specific DNA footprints in different tissues of wheat. ATAC-seq has now been widely used to explore chromatin accessibility, providing assistance for mining *cis*-regulatory elements. Since chromatin accessibility usually reflects the location of active *cis*-regulatory elements in the genome, the differentially OCRs in the two tissues reflect the *cis*-regulatory elements that vary between tissues, which may explain the differential expression of genes in different tissues. Differences in chromatin accessibility in different tissue type also offer new insights into explaining tissue specificity.

## Conclusion

In conclusion, by screening commonly used nuclei extraction buffers and combining fluorescence activated nuclei sorting, we have established a robust and broadly applicable method for wheat ATAC-seq experiment. Meanwhile, a flexible and easy-to-use ATAC-seq data analysis pipeline was also developed. We presented high quality data from hard-to-harvest young spike and ovary of wheat. The method we proposed here has potential to be adapted to other plant species. It is conducive to obtaining open chromatin information for mining *cis*-regulatory elements in plants.

## Data availability statement

The original contributions presented in the study are publicly available. This data can be found here: NGDC, CRA006423.

## Author contributions

WY conceived and designed the study. CH and DC developed the online pipeline for data analysis. XW and CC conducted the experiments. XW, CC, and CH analyzed the data and drew graphs. XW, CH, and WY wrote the manuscript. All authors read and approved the final manuscript.

## Funding

This work was supported by the National Key Research and Development Program of China (2020YFE0202300) and the Fundamental Research Funds for the Central Universities (2662020ZKPY002).

## Acknowledgments

We sincerely thank the computing platform of the National Key Laboratory of Crop Genetic Improvement in HZAU for providing the computational resources.

## Conflict of interest

The authors declare that the research was conducted in the absence of any commercial or financial relationships that could be construed as a potential conflict of interest.

## Publisher’s note

All claims expressed in this article are solely those of the authors and do not necessarily represent those of their affiliated organizations, or those of the publisher, the editors and the reviewers. Any product that may be evaluated in this article, or claim that may be made by its manufacturer, is not guaranteed or endorsed by the publisher.
