# Supplementary material for: Mapping open chromatin by ATAC-seq in bread wheat
Source: Front Plant Sci. 2022 Nov 16;13:1074873. doi: 10.3389/fpls.2022.1074873 (PMC9709403; doi:10.3389/fpls.2022.1074873)
Supplement: Supplementary file 1 [file DataSheet_1.docx]

***Supplementary Material***

# Supplementary Figures and Tables

- 1. **Supplementary Figure**


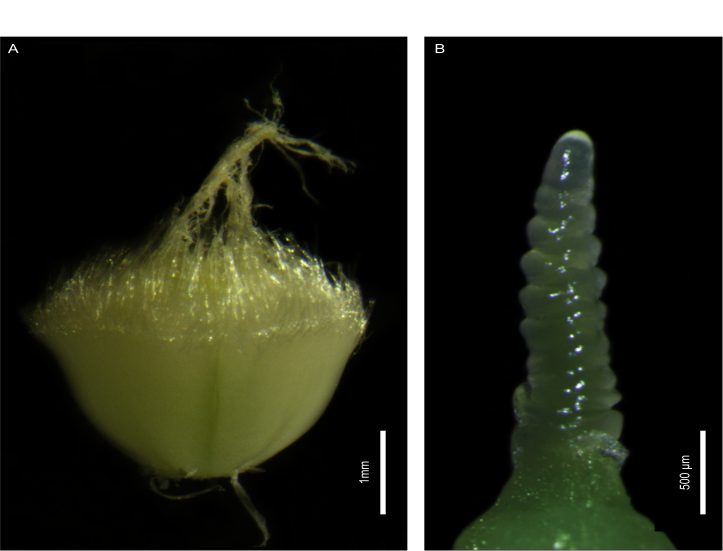


**Supplementary Figure 1.** Tissues used to produce ATAC-seq data. **(A)** Young spike at the double-ridge stage, about 2mm in length. **(B)** Ovary at two days after the middle spikelet flowers.

- 1. **Supplementary Tables**

**Supplementary Table 1.** PCR amplification primer sequences for FANS-ATAC-seq experiments

| **Primer name** | **Sequence** |
| --- | --- |
| PCR Primer I5-1 | AATGATACGGCGACCACCGAGATCTACACACATGCATTCGTCGGCAGCGTCAGATGTG |
| PCR Primer I5-2 | AATGATACGGCGACCACCGAGATCTACACACCATAGGTCGTCGGCAGCGTCAGATGTG |
| PCR Primer I7-1 | CAAGCAGAAGACGGCATACGAGATTGGTTCGAGTCTCGTGGGCTCGGAGATGT |
| PCR Primer I7-2 | CAAGCAGAAGACGGCATACGAGATCCAATTGCGTCTCGTGGGCTCGGAGATGT |

*Note:* The red fonts are the index sequences.

**Supplementary Table 2.** Alignment ratio of ATAC-seq data to wheat genome (ChrNu) , chloroplast (ChrC) and mitochondrial (ChrM) DNA

| **Sample name** | **Tissue** | **ChrNu** | **ChrC** | **ChrM** | **Peak numbers** |
| --- | --- | --- | --- | --- | --- |
| spike_rep1 | spike | 94.18% | 0.54% | 2.38% | 70766 |
| spike_rep2 | spike | 91.39% | 0.60% | 2.65% |  |
| ovary_rep1 | ovary | 94.87% | 0.08% | 0.28% | 86041 |
| ovary_rep2 | ovary | 94.49% | 0.10% | 0.35% |  |
